# Pyæmia, or Leukæmia

**Published:** 1863-12

**Authors:** Geo. R. Weeks

**Affiliations:** Surgeon of U. S. Vol., in charge of Church Hospital


### PYÆMIA, OR LEUKÆMIA.

By GEO. R. WEEKS, Surgeon of U. S. V.,
*In Charge of Church Hospital.*

*Church U. S. Military Hospital, Memphis, Tenn., Sept. 8th,* 1863.

Having recently been much interested in various pathologi cal conditions resulting in Pyæmia, Leukæmia, or Leucocythæmia, and believing that I have observed some facts of interest in this disease, I submit the following observations, taken at the bedside, with a view of calling attention to the true nature and conditions found in those affected by this particular grade of action. If I can be able to contribute in the least degree to this end, I shall feel myself amply rewarded. I believe it closely allied to *Hospital Gangrene*, and blood poisoning, and generally has appeared to be the sequence of these diseases.

The ideas that I shall present were taken from the observation and treatment of *one hundred and seventy-five* cases of Hospital Gangrene, one hundred and fifteen of which were observed and reported on by me, in Louisville, Ky. Six occurred in the 17th Army Corps Hospital, at Vicksburg, Miss., of which I had charge, and fifty-four at this Hospital, during the month of August, with what results will be shown by the following table.

Summary of Cases Treated by Bromine, Locally, and Their Results,

| No. Treated. | No. Recovered. | Died. | Died of: Gangrene. | Pyæmia. | Thrombus & Sepsis of the Blood. | Mechanical Pneumonia. | Cellulitis. | Diarrhœa. | Flesh Wounds. | Flesh Wounds, Bone Involved. | Average time of Arrest. Days. |
|---|---|---|---|---|---|---|---|---|---|---|---|
| 146 | 134 | 12 | | 3 | 5 | 2 | 1 | 1 | 104 | 42 | 4.26 |
| Summary of Cases Treated Without Bromine, and Their Result. | | | | | | | | | | | |
| 29 | 20 | 9 | 4 | 3 | 1 | 1 | | | 24 | 5 | 18.8 |

Fifty-three of these were treated locally by pure bromine, and the average time of arrest was 1.92 days, seldom requiring a second application. Ninety-three were treated by comp. bals. of bromine, average time being 8.66 days; and twenty-nine cases were treated by the usual methods, the average time of arrest was 18.8 days. All had the same constitutional treatment, and were alike situated in other respects.

The facts noticed during the treatment of these cases point to the following views with regard to its pathology. That Hospital Gangrene is produced by a *specific animal poison*, and that it only enters the system by inoculation upon a traumatic surface. That for a variable time after it is deposited upon or in the sore, it is local in character, after which it enters the blood through the agency of the absorbent system, and changes its constituents both vitally and chemically. I have designated this the stage of Toxæmia, in which the blood is morphologically changed, the result being thrombus, which in my opinion, is the plastic portion of the blood, aggregated and semi-organized, necessarily rendering the remainder aplastic and of little use to the economy in the work of repair. I have many morbid specimens now in my possession, that clearly establish the truth of these propositions, in illustration of which allow me to cite a few cases.

Adam Brangle, a private of 1st U. S. Inft'y, was brought to the 17th Army Corps Hospital, at Vicksburg, Miss., with gangrene of stump of right arm, which had been amputated several days previous to his admission. Upon examining him, I

diagnosed Mechanical Pneumonia, of right lung. He complained of pain in the heart, and over lower lobe of right lung, which then gave a flat sound, with dulness extending upward and with entire loss of vesicular resonance. No crepitus was heard in the part or above it.

I treated the arm locally, with pure bromine, which arrested the gangrene promptly, and the sore continued to granulate feebly until near death. The pulmonary difficulty continued upward until the entire right lung was involved and half of the left, when he died. I examined him carefully, and noticed his symptoms three times a day, myself, during the time he was under my care, being much interested in his condition. Autopsy twelve hours after death. The right lung was very much enlarged, and so heavy that it readily sank in water, so large that it filled the right side of the thorax entirely. Upon section it presented innumerable dark colored points of all sizes, which were traced back to where they could unmistakably be seen to be in the pulmonary artery. I hazard little by saying that very nearly all the vessels in the right lung were occluded by thrombi. Also a portion of the left was in the same condition, and in none of them was there any signs of a retrograde movement, or softening. This was a rapid case, and one that finely illustrates the mode of dying, which was undoubtedly from obstruction of a purely mechanical nature, so far as the lungs were concerned, producing an excess of carbonic acid in the blood, and a deficiency of oxygen from pressure on the vesicles and obliteration of air space. This is the first stage, or one necessary link in the pyæmic process.

2nd. James A. Beaver, private, co. A, 33d Iowa Infty Vol., was admitted to this Hospital July 30th, 1863, with gangrene of the ankle, involving the ankle joint and instep. I examined him August 8th, and diagnosed thrombus and septic poisoning. I made an unfavorable prognosis. Gangrene had been present since July 17th, and was promptly arrested by the use of bromine, locally, August 12th, but no repair was established. The wound kept clean and had no fœtor. He died August 18th. Autopsy ten hours after death.

The pulmonary vessels were filled with a dark colored, semifluid substance, and at points portions of unsoftened thrombi yet remained. In some of the larger vessels thrombus was still entire, in others, where the pressure was greatest, softening had commenced. Softening was also observed in the parenchyma, always most advanced in that part nearest the vessel. I found a large thrombus in the left side of the heart, more than half of it softened, and softening of the walls of the heart, with which it came in contact. It looked of a purple color, and had a gangrenous odor, and was easily broken down between the thumb and finger upon moderate pressure. I take it that this case points to the transition stage from thrombus to true pyæmia, and marks the process quite clearly.

Death perhaps was produced by ichoræmia, depraved or perverted nutrition, caused by the septic condition of the blood, the morbid element of which was indebted to softened thrombi, and the product returned to the general circulation rendered it more poisonous.

3d. J. H. Sutton, private, co. I, 36th Ind. Infty Vol., passed through both the above mentioned stages, and died of the next or of true pyæmia. Metastatic abscesses were formed in the lower lobe of right lung, of all sizes from a millet seed to that of a walnut, and also thrombi, entire and softening, forming new centres for abscesses. The man had been sick a long time, and had gangrene in popliteal space, which had been arrested previous to death some considerable time, but not in time to prevent ichoræmia.

I cite these three cases as marking the stages leading to the state of pyæmia distinctly and clearly. I have endeavored to present the facts as I have observed them, and let the results follow. Post-mortem appearances are nature's teachings, before whose power our most subtile theories and ingenious hypotheses must bow in humble submission. I have examined all that died, in every one of which I have found thrombus in the heart and lungs, in some of the stages before mentioned, in consequence of which I am inclined to look upon them as a necessary condition. I am fully persuaded that Virchow is right in calling it leukæmia, as I shall endeavor to prove hereafter. These remarks are not only true of gangrene, but are equally so of all that class of diseases arising from blood-poisoning, in which also ichoræmia secondarily plays an important part. I am not sure but that it will enable us to explain all the phenomena presented. The accompanying symptoms are very analogous to those arising from the bite of a venomous serpent, and perhaps what will cure one will arrest the other. I said in the commencement that, primarily, I believe it to be local, and that it passed into the blood by the agency of the absorbents, when toxæmia was produced; but how is this brought about? In what manner are these changes produced? I answer, 1st, By contact of the morbid agent with the traumatic surface. 2d, Transformation of cell contents, which I am inclined to believe is the result of oxydation. 3d, Absorption of the ichorous product, which causes a retrograde movement in the part and also in the constituents of the blood, which I think is chemico-vital in character. The corpuscular change is quite apparent by the aid of the microscope. The red corpuscle loses its hæmatine, which becoming granular, is deposited in a circle, and aggregated around the outside of the field. The white corpuscles are very abundant and occupy the whole surface. Chrystals of ammoniacal salts are formed in abundance, and an occasional fungi may be seen. Virchow teaches that it is not possible for substances, or rather particles of matter to pass through the lymphatic glands, as in the case of cinnabar and tattooing, they are arrested and detained there. I cannot say how it is accomplished, but of the fact that the morbific agent does pass the glands, I am well satisfied, and as these are the last row of sentinels to contend against, I can see no reason why it should not proceed onward to the blood. I have a case in point.

James Scott, private, co. A, 1st Mo., died at this Hospital, on Sept. 3d, in whose thoracic duct I found a well-developed thrombus. I sent it to Surgeon Brinton, U. S. V., Washington. It was about four inches long, and largest in that portion that occupied the receptaculum chyli. It is the first case that I have ever seen or heard of. The glands were enlarged, indurated, and of a dark color, as if filled with pigment granules, which I very much suspect they were. The ichor undoubtedly had passed, as its effects were demonstrated by thrombus in the duct. If so the whole process corresponds very nearly to Virchow's idea of their function. This case conclusively presents three very important facts, viz.: That the poison, whatever it is, passes through the lymphatic gland; that it is capable of producing thrombus upon the other side, and that the morbid element does not reside in the pigment but in its ichor. I am of the opinion that this settles a vital point in the pathology of leukæmia.

The death of the parts afflicted by gangrene is a molecular one, and I believe should occupy an intermediate position between ulceration and mortification. It is always accompanied by asthenia.

Of the cases observed here during the month, the average temperature of the body was 89° Fahrenheit, and the number of pulsations were 101, which significantly pointed to the manner in which they die, and to the indications for treatment. In he formation of thrombus I have invariably found them of the shape of the vessels, upon the right side, and globular upon the left, and in the latter seldom extending into the vessels. I have one of the left auricle now, weighing 482 grains, and one of the right side, representing every branch of the pulmonary artery, which had its attachment to the right ventricle.

It will be inferred from what I have said previously, that I regard the disease as curable, before its consequences are constitutionally developed. This is exactly what I at present believe. I can see no reasonable prospect of reaching it afterwards and would not know how to make a rational prescription, with my present views of its pathology, other than support. Upon the appearance of thrombus in the *heart* and

*lungs*, which is easily detected by the physical signs, hope with me ceases. Some may recover but I have not seen them. The rational remedies indicated I believe to be, oxygen, fluorine, chlorine, bromine, etc. I expect much from the judicious application of the former, and shall try it as soon as opportunity offers. Fluorine is too dangerous. Chlorine can only be obtained in a fluid state, under great pressure, or in the form of mechanical mixture, hence the selection of bromine, which is in the best form, and easiest of application, and over which condition I believe it to have a specific influence. I am of the opinion that it will arrest gangrene wherever it can be brought in contact with all the pulp or pultaceous matter. This is very difficult to do in the burrowing form of the disease, and also from the nature of some wounds, it is nearly impossible to reach every part with the remedy.

I have been industriously seeking an agent that would destroy the poison in the blood, or counteract its effects, but up to the present time have found none. I first tried the hyposulphites, then bromine by inhalation, and then internally. I have observed no beneficial effects from the hyposulphites, or the inhalation of bromine, but am inclined to believe that the internal administration of bromine, is of some value, how much I am unable yet to determine. I am now putting it to a severe test, in all the cases under my care, and noticing its effects closely with a view to settling this point. I am using the following formula: ℞. Bromine, Comp. Sol. of, ʒ ij; Aqua Distillata, ℥ ij; Syr. Simplex, ℥ ij; Mix. S. Teaspoonful every six hours.

I am favorably impressed with the remedy, but will not be able to speak with certainty until I have tried it for a longer period, and observed its effects more closely.

